# A retrospective study of neoadjuvant FOLFIRINOX in unresectable or borderline-resectable locally advanced pancreatic adenocarcinoma

**DOI:** 10.1186/1471-2407-12-199

**Published:** 2012-05-29

**Authors:** Peter J Hosein, Jessica Macintyre, Carolina Kawamura, Jennifer Cudris Maldonado, Vinicius Ernani, Arturo Loaiza-Bonilla, Govindarajan Narayanan, Afonso Ribeiro, Lorraine Portelance, Jaime R Merchan, Joe U Levi, Caio M Rocha-Lima

**Affiliations:** 1 Department of Medicine, Division of Hematology/Oncology, University of Miami/Sylvester Comprehensive Cancer Center, Miami, FL, USA; 2 Institution: Department of Medicine, Division of Hematology/Oncology, University of Miami/Sylvester Comprehensive Cancer Center, Miami, FL, USA; 3 Advanced Oncology Center of Hospital São José, São Paulo, Brasil; 4 Department of Medicine, Division of Hematology/Oncology, University of Miami/Sylvester Comprehensive Cancer Center, Miami, FL, USA; 5 Department of Medicine, University of Miami/Sylvester Comprehensive Cancer Center, Miami, FL, USA; 6 Department of Medicine, Division of Hematology/Oncology, University of Miami/Sylvester Comprehensive Cancer Center, Miami, FL, USA; 7 Department of Radiology, University of Miami/Sylvester Comprehensive Cancer Center, Miami, FL, USA; 8 Department of Medicine, Division of Gastroenterology, University of Miami/Sylvester Comprehensive Cancer Center, Miami, FL, USA; 9 Department of Radiation Oncology, University of Miami/Sylvester Comprehensive Cancer Center, Miami, FL, USA; 10 Department of Medicine, Division of Hematology/Oncology, University of Miami/Sylvester Comprehensive Cancer Center, Miami, FL, USA; 11 Department of Surgery, University of Miami/Sylvester Comprehensive Cancer Center, Miami, FL, USA; 12 Department of Medicine, Division of Hematology/Oncology, University of Miami/Sylvester Comprehensive Cancer Center, Miami, FL, USA

**Keywords:** Pancreatic ductal carcinoma, neoadjuvant therapy, surgery, radiation therapy

## Abstract

**Background:**

5-fluorouracil, leucovorin, irinotecan and oxaliplatin (FOLFIRINOX) is superior to gemcitabine in patients with metastatic pancreatic cancer who have a good performance status. We investigated this combination as neoadjuvant therapy for locally advanced pancreatic cancer (LAPC).

**Methods:**

In this retrospective series, we included patients with unresectable LAPC who received neoadjuvant FOLFIRINOX with growth factor support. The primary analysis endpoint was R0 resection rate.

**Results:**

Eighteen treatment-naïve patients with unresectable or borderline resectable LAPC were treated with neoadjuvant FOLFIRINOX. The median age was 57.5 years and all had ECOG PS of 0 or 1. Eleven (61 %) had tumors in the head of the pancreas and 9 (50 %) had biliary stents placed prior to chemotherapy. A total of 146 cycles were administered with a median of 8 cycles (range 3-17) per patient. At maximum response or tolerability, 7 (39 %) were converted to resectability by radiological criteria; 5 had R0 resections, 1 had an R1 resection, and 1 had unresectable disease. Among the 11 patients who remained unresectable after FOLFIRINOX, 3 went on to have R0 resections after combined chemoradiotherapy, giving an overall R0 resection rate of 44 % (95 % CI 22–69 %). After a median follow-up of 13.4 months, the 1-year progression-free survival was 83 % (95 % CI 59-96 %) and the 1-year overall survival was 100 % (95 % CI 85-100 %). Grade 3/4 chemotherapy-related toxicities were neutropenia (22 %), neutropenic fever (17 %), thrombocytopenia (11 %), fatigue (11 %), and diarrhea (11 %). Common grade 1/2 toxicities were neutropenia (33 %), anemia (72 %), thrombocytopenia (44 %), fatigue (78 %), nausea (50 %), diarrhea (33 %) and neuropathy (33 %).

**Conclusions:**

FOLFIRINOX followed by chemoradiotherapy is feasible as neoadjuvant therapy in patients with unresectable LAPC. The R0 resection rate of 44 % in this population is promising. Further studies are warranted.

## Background

Pancreatic cancer carries a dismal prognosis with a 5-year overall survival rate of 6 %. It is the fourth leading cause of cancer death in the USA with an estimated 37,660 deaths in 2011 [1]. Surgery remains the best chance for cure but only 10-20 % of patients will be candidates for curative resection at diagnosis. Up to 40 % of newly diagnosed patients will present with locally advanced, unresectable disease because of vascular encasement, and consensus criteria now exist to clearly define this group [2]. Up to one-third of patients who initially have locally advanced, unresectable disease may become resectable after neoadjuvant therapy [3]. These patients can have comparable survival to those who were initially resectable.

Systemic chemotherapy for advanced pancreatic cancer has generally been disappointing. The traditional gemcitabine-based therapies result in low response rates and short survival times in the range of 6 months. Interest in systemic chemotherapy for this disease was recently rejuvenated with the advent of the FOLFIRINOX combination (5-fluorouracil, leucovorin, irinotecan and oxaliplatin) [4]. In the randomized phase III ACCORD-11 trial, FOLFIRINOX was shown to be superior to single-agent gemcitabine in response rate (RR), progression-free survival (PFS) and overall survival (OS). The RR in the FOLFIRINOX arm was 32 % versus 9 % in the gemcitabine arm, and this translated into an improvement in PFS to 6.4 months (versus 3.3 in the control arm, p < 0.0001) and OS of 11.1 months (versus 6.8 months in the control arm, p < 0.0001). This trial only enrolled patients with metastatic pancreatic cancer. In the setting of LAPC, a good tumor response is a desirable goal since tumor shrinkage away from vascular structures may lead to resectability.

Radiation therapy may be beneficial in patients with LAPC and combined chemoradiotherapy (CCRT) has been tested in this population. Although 5-FU-based CCRT is commonly used in this setting, gemcitabine-based CCRT is safe and effective [5,6]. The best sequence of treatment administration has not been established. Prospective randomized trials are ongoing to evaluate whether there is a survival benefit of CCRT versus chemotherapy alone. Retrospective data suggest superiority of sequencing chemotherapy followed by chemoradiation [7]. The theoretical advantages of this approach include eradicating micrometastatic disease early on, and also identifying patients who progress early and develop metastases during initial chemotherapy (an estimated 25-30 % of patients), thus avoiding morbidities associated with radiotherapy in this group of patients.

Our group at the University of Miami adopted the approach of induction chemotherapy followed by CCRT in patients with unresectable or borderline resectable LAPC. Although the phase III results supporting the use of FOLFIRINOX in pancreatic cancer were only recently presented in 2010, the initial FOLFIRINOX randomized phase II trial (reported in 2007) showed a similarly high RR [8]. After these promising results were presented, we began using this regimen in patients with borderline resectable (BR) or unresectable (UR) LAPC. The aim of this retrospective study was to define the efficacy and toxicities of this regimen in LAPC, especially in patients with pancreatic head tumors and biliary obstruction – a group that was relatively underrepresented in the ACCORD-11 trial.

## Patients and Methods

We performed a retrospective analysis at the University of Miami Sylvester Comprehensive Cancer Center and Jackson Memorial Hospital in Miami, Florida of all patients with LAPC who received first-line treatment with FOLFIRINOX. Patients were identified by searching the pancreatic cancer database which was approved by the University of Miami Institutional Review Board (IRB). This IRB-approved database provided a waiver of the requirement for informed consent for retrospective studies and allowed for publication of de-identified data.

The medical, radiation and surgical oncologists in the group developed an algorithm for uniform treatment of this group of patients as follows. Patients were selected for treatment with FOLFIRINOX if they had a histological or cytological diagnosis of pancreatic adenocarcinoma, an Eastern Cooperative Group performance status (ECOG PS) of 0 or 1, adequate organ function, and UR/BR LAPC. For patients who presented with biliary obstruction, adequate biliary drainage was required prior to initiation of this chemotherapy. The determination of resectability was made by multidisciplinary review. Since there is currently no internationally agreed-upon definition of borderline-resectable LAPC, we applied the American Hepato-Pancreato-Biliary Association/Society of Surgical Oncology/Society for Surgery of the Alimentary Tract (AHPBA/SSO/SSAT) criteria [2]. These criteria are summarized in Table 1. The data used to determine resectability included pretreatment contrast-enhanced CT scans (CECT's) and endoscopic ultrasound (EUS) in all patients and surgical exploration when available.

**Table 1:** Criteria used for determining resectability for non-metastatic pancreatic cancer

Category	Criteria^§^
Resectable	· No evidence of SMV and portal vein abutment, distortion, tumor thrombus or venous encasement
	· Clear fat planes around the celiac axis, hepatic artery and SMA
Borderline resectable	· Venous involvement of the SMV/portal vein demonstrating tumor abutment with or without impingement and narrowing of the lumen, encasement of the SMV/portal vein but without encasement of the nearby arteries, or short segment venous occlusion resulting from either tumor thrombus or encasement but with suitable vessel proximal and distal to the area of vessel involvement, allowing for safe resection and reconstruction.
	· Gastroduodenal artery encasement up to the hepatic artery with either short segment encasement or direct abutment of the hepatic artery, without extension to the celiac axis
	· Tumor abutment of the SMA not to exceed 180 degrees of the circumference of the vessel wall
Unresectable	· Major venous thrombosis of the portal vein or SMV extending for several centimeters
	· Circumferential encasement of the SMA, celiac axis or proximal hepatic artery

Abbreviations: SMA: Superior mesenteric artery; SMV: Superior mesenteric vein.

^§^Adapted from the American Hepato-Pancreato-Biliary Association/ Society of Surgical Oncology/ Society for Surgery of the Alimentary Tract (AHPBA/SSO/SSAT) criteria (reference 2).

Treatment with FOLFIRINOX proceeded with doses identical to the ACCORD-11 trial [4]. On day 1 of every 14-day cycle, oxaliplatin was administered at a dose of 85 mg per square meter, irinotecan at 180 mg per square meter, folinic acid at 400 mg per square meter, and 5-fluoruracil (5-FU) as a bolus of 400 mg per square meter. 5-FU was then administered as a continuous infusion of 2400 mg per square meter over 46 hours. Filgrastim was at the discretion of the treating physician. Treatment continued until progression of disease, intolerable toxicity or maximum response. Toxicities were assessed at every fortnightly visit and graded according to the National Cancer Institute Common Terminology Criteria for Adverse Events, version 4 [9]. Criteria for dose modification were similar to those described in the ACCORD-11 trial. Response assessment by CECT's were performed every 8 weeks on therapy or sooner if progression was suspected by symptoms or rising CA19-9.

After every CECT scan during treatment, each patient’s case was reviewed at a multidisciplinary conference to determine whether the reason for defining the patient as UR or BR had improved. Since size was not the only criteria used in this evaluation, traditional response criteria (such as RECIST) were not employed. For example, if a tumor was categorized as BR due to abutment of the SMA up to 180°, the extent of abutment was re-evaluated after every scan to determine if this was improving and if the patient would now be a surgical candidate. If two consecutive scans during treatment showed similar findings with no improvement, this was considered to be the maximum response. Maximum tolerability was defined as the point when excessive toxicities warranted stopping FOLFIRINOX, even if a patient had not achieved their maximum response. Because we used an algorithm of real-time monitoring of response and toxicity, there was no predefined minimum or maximum number of cycles.

At maximum response or tolerability, patients who appeared to be resectable by imaging criteria were offered surgical exploration and resection (within 6-8 weeks after chemotherapy) followed by postoperative CCRT. Patients who remained unresectable at maximum response or tolerability of FOLFIRINOX were offered CCRT. For radiation sensitization, patients received concurrent gemcitabine at 600 mg per square meter per week. Intensity-modulated radiation therapy (IMRT) was delivered in a standard fashion to a total dose of 50.4 Gy in 28 fractions. At the end of CCRT, patients were re-evaluated with CECT's to determine resectability. Post-CCRT treatment was left to the discretion of the treating physicians.

The primary endpoint for this analysis was the R0 resection rate. An R0 resection was defined as at least 1 mm free margins. An R1 resection was defined as tumor within 1 mm from the closest margin. Secondary endpoints included safety, tolerability, overall survival and progression-free survival. All patients who received at least one cycle of FOLFIRINOX were included in the analysis. PFS and OS were calculated as follows; PFS was defined as the duration in months from the date of the first cycle of FOLFIRINOX until the date of documented progression, recurrence or death, whichever was sooner. OS was defined as the duration in months from the date of the first cycle of FOLFIRINOX until the date of death from any cause. Patients known to be alive were censored at the time of last contact. PFS and OS were estimated by the Kaplan–Meier method with corresponding two-sided 95 % CI's for survival proportions based on Greenwood's variance and the log-transform method [10]. Descriptive statistics were also used to summarize data.

## Results

Between 5/2008 and 5/2011 we administered FOLFIRINOX to 18 treatment-naive patients with UR/BR LAPC. The baseline characteristics of these patients are summarized in Table 2. One patient was defined as unresectable at the time of exploratory laparotomy and the remaining patients fulfilled criteria for UR/BR disease based on CECT and EUS findings. One patient who had splenic vein encasement was potentially resectable according to the AHPBA/SSO/SSAT criteria but underwent neoadjuvant therapy because the tumor was large and ill defined and the multidisciplinary group felt that the patient would benefit from neoadjuvant therapy. Among the 11 patients who had tumors at the head of the pancreas, 9 had metallic stents placed to relieve biliary obstruction and the other 2 had double-bypass operations which included a choledochojejunostomy. All patients who had stents or bypass procedures achieved normalization of their serum bilirubin levels prior to the start of chemotherapy.

**Table 2:** Patient characteristics, n = 18

n (%)	
**Age**	
Median	57.5 years
Range	41 – 73 years
**Sex**	
Male	10 (56 %)
Female	8 (44 %)
0	8 (44 %)
1	10 (56 %)
**Pancreatic tumor location**	
Head	11 (61 %)
Uncinate process	3 (17 %)
Body	2 (11 %)
Tail	2 (11 %)
**Biliary stent**	
Yes	9 (50 %)
No	9 (50 %)
**Serum bilirubin level prior to chemotherapy**	
Median	0.35 mg/dl
Range	0.1 – 1.2 mg/dl
**Basis for unresectability**	
**Borderline resectable**	
SMV encased (short segment)	3 (17 %)
SV encased	1 (6 %)
SMV encased (long segment)	1 (6 %)
SMA encased	3 (17 %)
HA encased	2 (11 %)
Celiac trunk encased	3 (17 %)
Confluence of PV, SV and SMV encased	5 (28 %)

Abbreviations: HA: Hepatic artery; PV: Portal vein; SMA: Superior mesenteric artery; SMV: Superior mesenteric vein; SV: Splenic vein.

**Figure 1:**
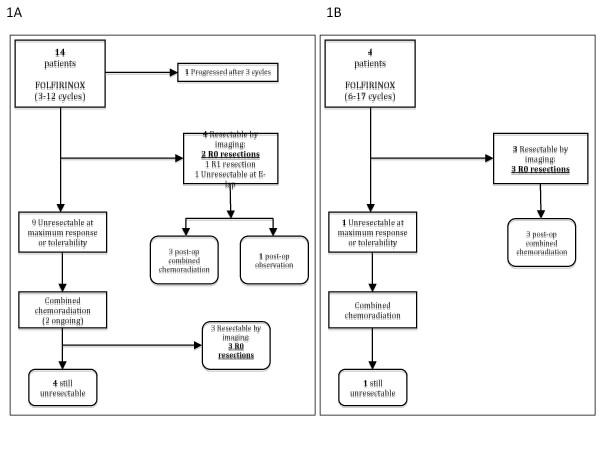
Patient flowchart for patients assessed as having unresectable disease (panel 1A, n = 14), and for those assessed as having borderline resectable disease (panel 1B).

Figure 1 shows the patient flow through the treatment algorithm. After FOLFIRINOX therapy, 7 patients had disease that was radiologically assessed as being potentially resectable. Five of these went on to have R0 resections, one R1 and one was grossly unresectable at exploration. Among the remaining 11 patients, one developed progression after 3 cycles and 10 went on to receive CCRT. Among these 10 patients, 2 were ongoing on CCRT at the time of this report; 5 remained with unresectable disease; and 3 were radiologically assessed as being potentially resectable after CCRT and all 3 went on to have R0 resections. The overall R0 resection rate was 8 out of 18 or 44 % (95 % CI 22-69 %) using this sequential combined modality approach. Among the 8 patients with successful R0 resections, the median number of FOLFIRINOX cycles delivered was 6 (range 5-17). In these patients, the reasons for UR/BR at baseline were SMV encasement in 2 patients, SMV/portal vein confluence encasement (2 patients), SMA encasement >180 degrees (1 patient), hepatic artery encasement (2 patients) and splenic vein encasement (1 patient).

**Figure 2:**
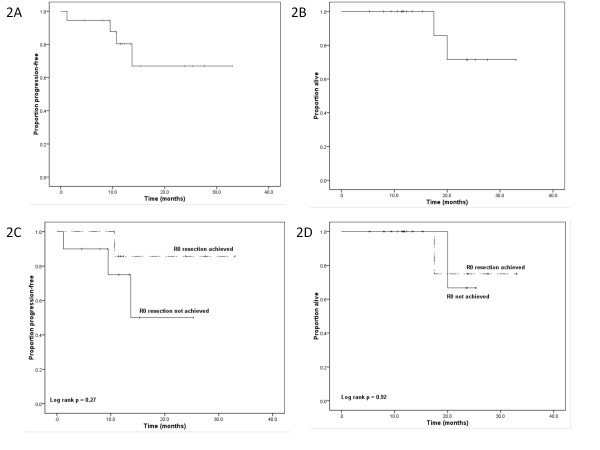
Kaplan-Meier survival curves: Panel 2A - progression-free survival for all patients; Panel 2B – overall survival for all patients; Panel 2 C - progression-free survival stratified by resection status; Panel 2D – overall survival stratified by resection status.

With a median follow-up of 13.4 months, 2 patients have died and 2 others are alive with progression of disease. One of the patients who died had an R0 resection and progressed 11 months after surgery. The Kaplan-Meier estimated median PFS and OS have not been reached. The 1-year PFS was 83 % (95 % CI 59-96 %) and the 1-year OS was 100 % (95 % CI 85-100 %). The estimated 1-year PFS was not significantly different for the patients who achieved R0 resections (88 %; 95 % CI 47-100 %) versus those who did not (80 %; 95 % CI 44-94 %, p = 0.27). Figure 2 shows the Kaplan-Meier plots for PFS and OS, including a stratification by resection status.

A total of 146 cycles of FOLFIRINOX were delivered among the 18 patients treated, with a median of 8 cycles per patient. Twenty-five cycles (17 %) were given at a reduced dose, with 7 of these (5 %) being given without irinotecan in one patient. Table 3 summarizes the treatment delivery. All patients experienced at least one grade 1 or 2 adverse event and 8 patients (44 %) experienced at least one grade 3 or 4 adverse event. The adverse event profile is shown in Table 4. There were no significant differences in the toxicity profile for patients who had biliary stents versus those who did not. Filgrastim was used in 89 % of patients, and was started prophylactically from cycle 1 in 50 %; in spite of this, 22 % of patients had grade 3 or 4 neutropenia and 17 % had neutropenic fever. No patient had to be taken off FOLFIRINOX for toxicity. Ten patients (56 %) had a treatment delay of at least one week due to toxicity (most commonly for neutropenia or neutropenic fever). There were no episodes of cholangitis during FOLFIRINOX in any of the 18 patients treated.

**Table 3:** Treatment delivery, n = 18

	n (%)
**Number of cycles**	
≤ 4	2 (11 %)
5-8	8 (44 %)
9-12	7 (39 %)
≥ 13	1 (6 %)
Median	8 cycles
Dose intensity*	
Total number of cycles	146
Number of cycles at full dose	121 (83 %)
Number of cycles at reduced dose	25 (17 %)
Number of cycles delayed by ≥ 1 week	13 (9 %)
5-Fluorouracil dose intensity	98 %
Irinotecan dose intensity	95 %
Oxaliplatin dose intensity	98 %

*Dose intensity is defined as the dose delivered divided by the full dose.

**Table 4:** Adverse events during chemotherapy (18 patients, 146 cycles)

	**Patients with biliary stent (n = 9)**		**Patients without biliary stent (n = 9)**		**All patients (n = 18)**	
**Adverse event**	**Grade 1/2**	**Grade 3/4**	**Grade 1/2**	**Grade 3/4**	**Grade 1/2**	**Grade 3/4**
	**n (%)**	**n (%)**	**n (%)**	**n (%)**	**n (%)**	**n (%)**
**Hematological**						
Neutropenia	3 (33 %)	**3 (33 %)**	3 (33 %)	**1 (11 %)**	6 (33 %)	**4 (22 %)**
Neutropenic fever	0	**2 (22 %)**	0	**1 (11 %)**	0	**3 (17 %)**
Anemia	6 (67 %)	**2 (22 %)**	7 (78 %)	**0**	13 (72 %)	**2 (11 %)**
Thrombocytopenia	3 (33 %)	**3 (33 %)**	5 (56 %)	**0**	8 (44 %)	**3 (17 %)**
**Non-hematological**						
Fatigue	6 (67 %)	**1 (11 %)**	8 (89 %)	**1 (11 %)**	14 (78 %)	**2 (11 %)**
Anorexia	3 (33 %)	**0**	1 (11 %)	**0**	4 (22 %)	**0**
Mucositis	1 (11 %)	**0**	1 (11 %)	**0**	2 (11 %)	**0**
Nausea	5 (56 %)	**0**	4 (11 %)	**0**	9 (50 %)	**0**
Vomiting	0	**0**	2 (22 %)	**0**	2 (11 %)	**0**
Diarrhea	3 (33 %)	**1 (11 %)**	3 (33 %)	**1 (11 %)**	6 (33 %)	**2 (11 %)**
Peripheral neuropathy	1 (11 %)	**0**	5 (56 %)	**0**	6 (33 %)	**0**
Alopecia	6 (67 %)	**0**	6 (67 %)	**0**	12 (67 %)	**0**

Among the 10 patients who went to surgery after FOLFIRINOX, 3 experienced significant postoperative complications. One patient developed *C. Difficile* colitis, gastroparesis and a subhepatic collection; one had an infected pancreatic bed collection requiring percutaneous drainage 3 times; and one had an infected splenic bed collection requiring percutaneous drainage. None of these patients received preoperative radiation. The median length of perioperative hospital stay for these 10 patients was 7 days (range 5 to 60).

## Discussion

FOLFIRINOX can be considered a new standard of care option in patients with metastatic pancreatic cancer with a good performance status. In a survey of the prescribing plans of American oncologists after the 2010 American Society of Clinical Oncology meeting (during which the ACCORD-11 phase III results were presented), 18 % planned to offer FOLFIRINOX to good performance status patients, compared with 0 % in the same survey in 2009 [11]. However, FOLFIRINOX has not been readily adopted by the oncology community in the US because of concerns for the tolerability of this regimen and the risk of cholangitis in patients with stents [12,13]. Some of these concerns were quieted with the publication of the full report of the ACCORD-11 trial, in which no cholangitis was observed and the rate of febrile neutropenia was only 5.4 % [4].

The current study represents the first published series of North American patients treated with FOLFIRINOX with locally advanced pancreas cancer. The regimen’s toxicity was for the most part reversible and manageable. We selected relatively young patients with excellent performance status and little or no comorbidities. We believe that patient selection for FOLFIRINOX cannot be overemphasized since we have anecdotally observed significantly poorer tolerability in older patients and those with major comorbidities. Furthermore, we believe that our practice of limiting the use of FOLFIRINOX to patients who had adequate biliary drainage also helped to lessen the toxicities since irinotecan depends on biliary elimination. Despite the use of prophylactic filgrastim in most patients, 3 (17 %) developed febrile neutropenia, including 2 who had biliary stents in place. The other common toxicities such as fatigue, nausea and diarrhea were manageable, and no patient had to be taken off chemotherapy due to intolerance. Although the full dose of the regimen could be delivered in 85 % of the administered cycles, treatment delays for toxicity were common. In an attempt to attenuate the toxicities of this regimen, the US cooperative groups are planning FOLFIRINOX studies without the 5-FU bolus and with mandatory prophylactic growth-factor support.

Our strategy of neoadjuvant FOLFIRINOX followed by CCRT in patients with UR/BR LAPC resulted in an R0 resection rate of 44 %, with 2 patients in the denominator still receiving CCRT. Although the follow-up is relatively short, only 2 patients have died and 7 of the 8 patients who underwent R0 resections remain progression-free at a median follow-up of 14.9 months. For patients who remained unresectable after FOLFIRINOX, CCRT was able to convert 3 out of 10 (30 %) to resectability, and may have provided some measure of disease control to the others whose disease remained unresectable. Interestingly, a recently reported study of gemcitabine-based CCRT (using the identical CCRT regimen used in our series) reported resections in only 2 out of 28 (7 %) patients [5]. Since our series is small and the event rate is low, it is difficult to draw definite conclusions. Nonetheless, we hypothesize that the use of induction treatment with FOLFIRINOX, followed by a non-cross resistant chemotherapeutic agent as a radiation sensitizer (gemcitabine) might be an advantageous strategy to improve response rates and resectability.

## Conclusions

Neoadjuvant therapy with FOLFIRINOX in patients with unresectable or borderline resectable locally advanced pancreatic cancer appears to be feasible in selected patients. Continued follow-up will determine if achieving an R0 resection will translate into a survival advantage in this group of patients. Further study of this sequential combined modality approach is warranted.

## Competing interests

The authors declare that they have no competing interest.

## Authors’ contributions

PJH, JM and CRL conceived the study, collected, analyzed and interpreted the data and drafted the manuscript. CK, JCM, ALB, GN, AR, LP, JRM and JUL made substantial contributions to data collection and analysis and drafting the manuscript. All authors approved the final version of the manuscript.

## Pre-publication history

The pre-publication history for this paper can be accessed here:

http://www.biomedcentral.com/1471-2407/12/199/prepub

## References

[B1] American Cancer Society Cancer Facts & Figures 2011 2011 American Cancer Society, Atlanta

[B2] Callery MP Chang KJ Fishman EK Talamonti MS William Traverso L Linehan DC Pretreatment assessment of resectable and borderline resectable pancreatic cancer: expert consensus statement Ann Surg Oncol 2009 16 7 1727 33 10.1245/s10434-009-0408-6 19396496

[B3] Gillen S Schuster T Meyer Zum Büschenfelde C Friess H Kleeff J Preoperative/neoadjuvant therapy in pancreatic cancer: a systematic review and meta-analysis of response and resection percentages PLoS Med 2010 7 4 e1000267 10.1371/journal.pmed.1000267 20422030 PMC2857873

[B4] Conroy T Desseigne F Ychou M Bouché O Guimbaud R Bécouarn Y Adenis A Raoul JL Gourgou-Bourgade S de la Fouchardière C Bennouna J Bachet JB Khemissa-Akouz F Péré-Vergé D Delbaldo C Assenat E Chauffert B Michel P Montoto-Grillot C Ducreux M Groupe Tumeurs Digestives of Unicancer; PRODIGE Intergroup. FOLFIRINOX versus gemcitabine for metastatic pancreatic cancer N Engl J Med 2011 364 19 1817 25 10.1056/NEJMoa1011923 21561347

[B5] Cardenes HR Moore AM Johnson CS Yu M Helft P Chiorean EG Vinson J Howard TJ Stephens AW Tai DF Loehrer PJ A Phase II Study of Gemcitabine in Combination With Radiation Therapy in Patients With Localized, Unresectable, Pancreatic Cancer: A Hoosier Oncology Group Study Am J Clin Oncol 2011 34 5 460 5 10.1097/COC.0b013e3181e9c103 20881474

[B6] Loehrer PJ Feng Y Cardenes H Wagner L Brell JM Cella D Flynn P Ramanathan RK Crane CH Alberts SR Benson AB Gemcitabine Alone Versus Gemcitabine Plus Radiotherapy in Patients With Locally Advanced Pancreatic Cancer: An Eastern Cooperative Oncology Group Trial J Clin Oncol 2011 29 31 4105 4112 10.1200/JCO.2011.34.8904 21969502 PMC3525836

[B7] Huguet F André T Hammel P Artru P Balosso J Selle F Deniaud-Alexandre E Ruszniewski P Touboul E Labianca R de Gramont A Louvet C Impact of chemoradiotherapy after disease control with chemotherapy in locally advanced pancreatic adenocarcinoma in GERCOR phase II and III studies J Clin Oncol 2007 25 3 326 31 10.1200/JCO.2006.07.5663 17235048

[B8] Ychou M Desseigne F Guimbaud R Ducreux M Bouché O Bécouarn Y Adenis A Montoto-Grillot C Luporsi E Conroy T Randomized phase II trial comparing FOLFIRINOX vs gemcitabine as first-line treatment for metastatic pancreatic adenocarcinoma. First results of the ACCORD 11 trial J Clin Oncol 2007 25 suppl 18 451617876011

[B9] National Cancer Institute Common Terminology Criteria for Adverse Events (CTCAE) Version 4.0 2009,

[B10] Collett D Modelling survival data in medical research 1994 Chapman & Hall, London

[B11] Bendell JC Britton S Green MR Immediate impact of the FOLFIRINOX phase III data reported at the 2010 ASCO Annual Meeting on prescribing plans of American oncology physicians for patients with metastatic pancreas cancer (MPC) J Clin Oncol 2011 29 Suppl 4 286

[B12] Kang SP Saif MW Three-drug combination regimen in pancreatic cancer treatment: are we there yet? JOP 2011 12 78 82 21386625

[B13] Kim R FOLFIRINOX: a new standard treatment for advanced pancreatic cancer? Lancet Oncol 2010 12 8 9 21050812 10.1016/S1470-2045(10)70237-0

